# The Mediating Role of Loneliness and Discrimination in The Association Between Adverse Childhood Experiences and Mental Health in The Working Population

**DOI:** 10.1192/j.eurpsy.2026.11318

**Published:** 2026-07-31

**Authors:** L. Blasco, V. Longo, A. Miranda-Mendizabal, J. Domènech-Abella, P. Cristóbal-Narváez

**Affiliations:** 1 Parc Sanitari Sant Joan de Déu, Sant Boi de Llobregat , Spain; 2 Department of Neurosciences, Biomedicine and Movement, Università degli Studi di Verona, Verona, Italy; 3 Teaching, Research and Innovation Department, Parc Sanitari Sant Joan de Déu, Sant Boi de Llobregat; 4 Instituto de Salud Carlos III, Centro de Investigación Biomédica en Red de Salud Mental. CIBERSAM, Madrid; 5 Department of Sociology, Universitat de Barcelona, Barcelona, Spain

## Abstract

**Introduction:**

Workplace loneliness has received growing attention in recent years due to its association with lower job satisfaction, reduced productivity, and poorer mental health. Beyond occupational settings, loneliness and perceived discrimination are increasingly recognised as critical social determinants, linked to reduced psychological well-being, impaired social functioning, and greater risk of depression and anxiety. At the same time, adverse childhood experiences are well-established precursors of poor adult mental health; however, the mechanisms underlying this association remain insufficiently understood.

**Objectives:**

The study examines the association between adverse childhood experiences and symptoms of depression and anxiety, and to assess the mediating role of loneliness and perceived discrimination in this relationship in the working population.

**Methods:**

A total of 3,000 employed individuals residing in Catalonia, aged 18 to 65, were surveyed using computer-assisted web interviews (CAWI). Logistic regression models were applied to identify factors associated with anxiety and depressive symptoms. Multivariate logistic regression models were further employed to examine the associations between trauma and mental health outcomes, as well as the mediating roles of loneliness and perceived discrimination.

**Results:**

Neuroticism, loneliness, and perceived discrimination were consistently associated with both depressive and anxiety symptoms. In the case of depression, lack of paid employment and the presence of chronic illness were significant risk factors, whereas sex, age, and marital status showed no associations. For anxiety, women, married individuals, and those with chronic illness were at greater risk, while older adults reported significantly fewer symptoms compared to younger adults. Education was not significantly related to either condition. Adverse childhood experiences were not directly associated with depression or anxiety; however, their effects were mediated mainly, with 75% of the association with depression and 76.9% of the association with anxiety explained by loneliness and discrimination. Specifically, loneliness accounted for 43.8% and discrimination for 31.3% of the mediated effect on depression, while both factors contributed equally (38.5% each) to the mediation of anxiety symptoms.

**Conclusions:**

The mediating role of loneliness and discrimination in the association between adverse childhood experiences and mental health highlights the importance of social processes in shaping vulnerability to depression and anxiety. Interventions that promote social connectedness and reduce stigma may help buffer the long-term impact of trauma.

**Disclosure of Interest:**

None Declared

